# Diagnostic Performance of Radiolabelled FAPI Versus [^18^F]FDG PET Imaging in Hepato-Pancreato-Biliary Oncology: A Systematic Review and Meta-Analysis

**DOI:** 10.3390/ijms26051978

**Published:** 2025-02-25

**Authors:** Rutger B. Henrar, Floris A. Vuijk, George L. Burchell, Susan van Dieren, Lioe-Fee de Geus-Oei, Geert Kazemier, Alexander L. Vahrmeijer, Daniela E. Oprea-Lager, Rutger-Jan Swijnenburg

**Affiliations:** 1 Department of Surgery, Amsterdam UMC Location Vrije Universiteit Amsterdam, de Boelelaan 1117, 1081 HV Amsterdam, The Netherlands; r.b.henrar@amsterdamumc.nl (R.B.H.); s.vandieren@amsterdamumc.nl (S.v.D.); g.kazemier@amsterdamumc.nl (G.K.); 2 Cancer Center Amsterdam, Imaging and Biomarkers, Van der Boechorststraat 6B, 1081 BT Amsterdam, The Netherlands; daniela.oprea-lager@radboudumc.nl; 3 Department of Surgery, Leiden University Medical Center, Albinusdreef 2, 2333 ZA Leiden, The Netherlands; f.a.vuijk@lumc.nl (F.A.V.); a.l.vahrmeijer@lumc.nl (A.L.V.); 4 Medical Library, Vrije Universiteit Amsterdam, de Boelelaan 1118, 1081 HV Amsterdam, The Netherlands; g.b.burchell@vu.nl; 5 Department of Radiology, Leiden University Medical Center, Albinusdreef 2, 2333 ZA Leiden, The Netherlands; l.f.de_geus-oei@lumc.nl; 6 Department of Medical Imaging, Radboud University Medical Center, Geert Grootplein Zuid 10, 6525 GA Nijmegen, The Netherlands

**Keywords:** meta-analysis, FAPI, FDG, PET/CT, hepato-pancreato-biliary cancer, pancreatic cancer, hepatocellular carcinoma, biliary tract cancer, liver metastasis

## Abstract

Radiolabelled fibroblast activation protein inhibitor (FAPI) tracers have the potential to overcome the limitations of 2-deoxy-2-[^18^F]fluoro-D-glucose ([^18^F]FDG) and improve the diagnosis and staging of hepato-pancreato-biliary (HPB) cancers. This study aims to compare the diagnostic performance of radiolabelled FAPI versus [^18^F]FDG PET imaging in HPB cancers. A systematic search of PubMed, Embase, Web of Science and Cochrane Library was performed to identify eligible studies on the diagnostic performance of FAPI PET for primary HPB tumours (hepatocellular carcinoma (HCC), pancreatic cancer (PC) and biliary tract cancer (BTC)) and for liver metastases of gastrointestinal origin. The diagnostic performance was defined as a combination of detection rate and semi-quantitative tracer uptake. A random-effects model was used to calculate the risk differences. In total, 28 studies were included. Histopathology was the reference standard for the primary tumour in 26 studies (93%). The detection rate of radiolabelled FAPI in comparison to [^18^F]FDG was significantly higher in HCC (0.33, 95% CI: 0.20–0.47 and 0.34, 95% CI: 0.23–0.45) and BTC (0.27, 95% CI: 0.11–0.43 and 0.28, 95% CI: 0.08–0.48), in the patient- and lesion-based analyses, respectively. In PC, no differences were observed. Radiolabelled FAPI outperformed [^18^F]FDG in the lesion-based detection of lymph node, liver and extra-hepatic metastases. In all HPB cancers, the mean SUVmax was significantly higher with radiolabelled FAPI compared to [^18^F]FDG. Molecular imaging with FAPI PET seems to have several benefits over [^18^F]FDG PET in HPB cancer diagnostics, with an overall higher tracer uptake, and higher detection rates in HCC and BTC.

## 1. Introduction

Hepato-pancreato-biliary (HPB) cancers affected an estimated 1.5 million individuals globally in 2020, representing the second leading cause of all cancer-related deaths [1]. The most prevalent types of primary HPB tumours are hepatocellular carcinoma (HCC), pancreatic cancer (PC, specifically pancreatic ductal adenocarcinoma (PDAC)) and biliary tract cancer (BTC). Additionally, the liver is a common site of distant metastases of gastrointestinal cancers, with colorectal liver metastases being the most prevalent [2]. Most patients suffering from HPB cancers are asymptomatic or have non-specific symptoms until an advanced stage of disease, posing a diagnostic challenge.

The current diagnostic work-up of HPB malignancies is based on a combination of the following anatomical imaging modalities: contrast-enhanced computed tomography, magnetic resonance imaging and (endoscopic) ultrasound. However, these modalities have several shortcomings. The detection of small tumoural lesions (<1 cm), the diagnosis of (lymph node) metastases and the differentiation between fibrotic, inflammatory or tumoural lesions, especially after neoadjuvant chemotherapy, are not accurate enough for adequate staging and proper surgical patient selection [3,4,5,6].

Recent developments in molecular imaging may fundamentally change the tumour staging. Hybrid positron emission tomography/computed tomography (PET/CT) with different radiotracers potentially improves the diagnosis, response assessment and treatment selection. The most widely used imaging tracer is 2-deoxy-2-[^18^F]fluoro-D-glucose ([^18^F]FDG), based on increased metabolism and the glucose-trapping mechanism in malignant and inflammatory cells [7]. Recent publications have provided evidence supporting the benefit of [^18^F]FDG PET/CT imaging in primary HPB cancers and metastases [8,9,10,11]. However, it is not implemented as standard imaging for HPB cancers, due to its inability to distinguish between malignant and inflammatory processes.

Radiolabelled quinoline-based fibroblast activation protein inhibitors (FAPIs) are promising novel PET tracers that target cancer associated fibroblasts (CAFs) through specific binding to fibroblast activation protein (FAP) [12,13]. The Heidelberg group developed the first gallium-68 labelled FAPI tracer and successfully tested it in various tumour types [14]. These radiopharmaceuticals possess theranostic potential when labelled with beta-emitting particles, such as lutetium-177 or yttrium-90 [15], leading to new applications of molecular imaging.

Primary HPB tumours are a promising group of malignancies for FAPI PET imaging, as they are histopathologically characterised by a high stromal content and recruitment of FAP-expressing CAFs [16,17,18]. The initial diagnostic results of FAPI PET imaging in HPB cancers showed potential for improved staging and diagnosis, therefore refining the selection of patients for curative intent treatment. In addition, previous studies have demonstrated that high expression of FAP is associated with a worse prognosis in various tumours, including PC and BTC [19,20,21]. This suggests that radiolabelled FAPI uptake could be used as an imaging biomarker to identify patients with unfavourable tumour biology. However, to assess the diagnostic performance and clinical impact of radiolabelled FAPI in HPB tumours, a direct comparison with [^18^F]FDG is necessary.

Therefore, the aim of this systematic review and meta-analysis was to compare the diagnostic performance of radiolabelled FAPI versus [^18^F]FDG PET imaging in primary HPB cancers (HCC, BTC and PC) and in liver metastases of gastrointestinal origin.

## 2. Materials and Methods

This systematic review and meta-analysis was developed and reported according to the Preferred Reporting Items for Systematic Reviews and Meta-Analyses (PRISMA) guidelines, and was registered in the PROSPERO database (registration ID: CRD42023415321) [22]. No institutional review board approval was needed, as published data were collected and analysed.

### 2.1. Literature Search and Study Selection

A systematic search was performed in four databases: PubMed, Embase, Clarivate Analytics/Web of Science Core Collection and the Wiley/Cochrane Library. The last search in these databases was performed on 25 February 2023. The search included keywords and free text terms for (synonyms of) ‘Pancreatic Neoplasms’, ‘Cholangiocarcinoma’, ‘Hepatocellular carcinoma’ or ‘Colorectal carcinoma’ combined with (synonyms of) ‘fibroblast activation protein’. A full overview of the search terms per database can be found in the Supplementary Materials (Supplementary Tables S1–S4). No limitations on date or language were applied in the search. The inclusion criteria were as follows: full text available in English, specification of the FAPI tracer and the focus of the study was the diagnostic performance of FAPI PET imaging (PET/CT or PET/MRI) in oncology, stating at least the diagnostic performance compared to other modalities or semi-quantitative tracer uptake metrics, i.e., maximum standardised uptake value (SUVmax) or target-to-background ratio (TBR). Studies with less than five patients with a primary liver, pancreas or biliary tract malignancy, or liver metastases of gastrointestinal origin were excluded. Reviews, animal studies, case reports and conference abstracts were also excluded. After removing duplicates, all remaining studies were screened individually on title and abstract by two reviewers (RH, FV) for potential inclusion. Potential studies were retrieved in full-text and assessed for eligibility. All eligible articles were then reviewed for quality using the Quality Assessment of Diagnostic Accuracy Studies-2 (QUADAS-2) criteria, scoring on four domains for risk of bias and on three domains for applicability, by the same researchers (RH, FV) [23]. Disagreements were solved in consensus.

### 2.2. Data Collection

The following study data were collected: general information on the author and study, patient characteristics, imaging protocol (type of tracer, injected activity, injection-scan interval), method of scan interpretation and blinding, type of reference standard used, semi-quantitative tracer uptake metrics and positive detection rate for each imaging modality, patient- and lesion-wise, of the primary tumour and lymph node, liver and extra-hepatic distant metastases. The diagnostic performance was defined as a combination of the detection rate, patient- and lesion-based detection and the semi-quantitative tracer uptake.

SUVmax was defined as the tumour uptake normalised to the patient distribution volume and the net injected activity. TBR represented the image-based uptake value of the target divided by the background uptake. Lymph node and extra-hepatic distant metastases of primary HPB cancers were collected. Liver metastases of gastrointestinal origin were collected as a separate entity. Liver metastases from general oncology studies were excluded. BTC included cholangiocarcinoma (intrahepatic, perihilar and distal) and gallbladder carcinoma. PC included all malignant pancreatic tumours.

### 2.3. Statistical Analysis

Data were summarised using descriptive statistics. A patient- and lesion-based comparison of the detection rate of radiolabelled FAPI and [^18^F]FDG PET imaging was performed for each oncological subgroup. For the effect size of the detection rate, the risk difference between radiolabelled FAPI and [^18^F]FDG was calculated with a 95% confidence interval (CI), with a random-effects model. In cases with no events in a study arm, a zero-cell-correction of 0.5 in all observations was made. Studies that did not compare radiolabelled FAPI to [^18^F]FDG or included patients with non-avid [^18^F]FDG lesions were excluded from the detection rate analysis. For the comparison of the effect size of the semi-quantitative metrics, the standardised mean difference (Cohen’s D) was calculated with a 95% CI for each oncological subgroup, with a random-effects model. If no mean and standard deviation were reported, an estimation was made based on the median and (interquartile) range using the method described by Wan [24]. A generalised mixed model was applied if the inversed variance method did not converge. Statistical heterogeneity between studies was calculated using the I^2^ statistic and the interpretation by Higgins et al. was followed [25]. A *p* value of 0.05 was used as a cut-off value for statistical significance. Statistical analyses were conducted under the supervision of a statistician (SvD) using SPSS Statistics (version 28) and RStudio (version 2022.02.3).

## 3. Results

### 3.1. Study Selection and Quality Assessment

**Figure 1:**
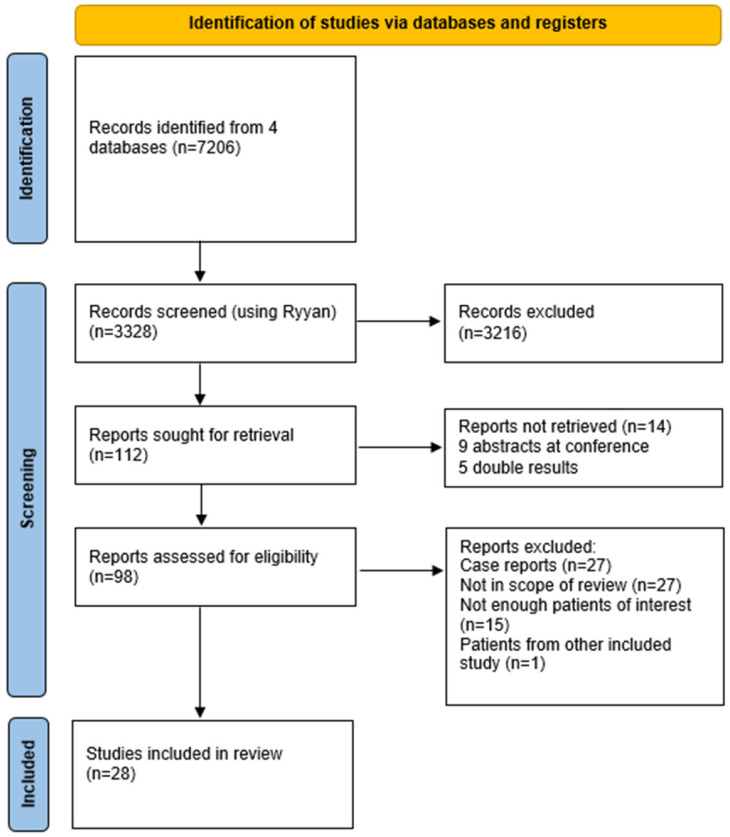
Flowchart of the selection of included studies.

**Figure 2:**
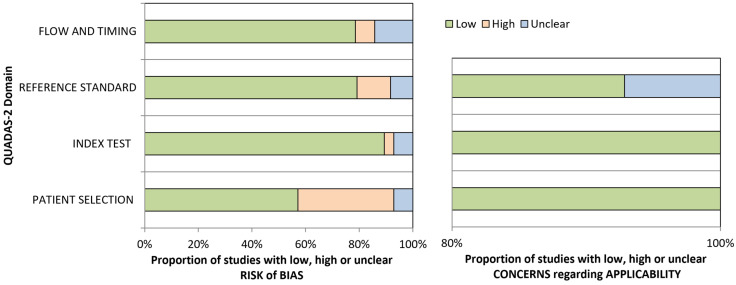
Visualization of the Quality Assessment of Diagnostic Accuracy Studies-2 (QUADAS-2) score of all included studies.

Out of 7206 records identified through our search strategy in four databases, 28 studies were included after screening the abstracts and conducting a full-text assessment of eligibility. The flowchart of the inclusion process is presented in Figure 1. The risk of bias was low in most domains (Figure 2). However, in the selection of patients, the risk of bias was high in 10 (36%) studies, due to selection criteria, such as inconclusive [^18^F]FDG PET findings, patients with a diagnostic challenge or specific metastases. The risk of bias of the reference standard was high in three (11%) studies. Concerns regarding applicability were very low in all domains.

### 3.2. Study Characteristics

An overview of the study characteristics is presented in Table 1 and Supplementary Table S5 [14,26,27,28,29,30,31,32,33,34,35,36,37,38,39,40,41,42,43,44,45,46,47,48,49,50,51,52]. In total, 1621 patients were included in this systematic review. The distribution of patients across the specific malignancies was as follows: 229 patients with PC, 128 with HCC, 94 with BTC and 176 with liver metastases of gastrointestinal origin. Most studies had a retrospective nature, were published in 2022 and were conducted in China as monocenter studies. Several studies had a common study registration and may have (partly) used data from previously published articles [27,28,29,30,31,32,33]. Six studies reported a potential conflict of interest of the authors [14,28,30,34,35]. The PET image acquisition protocols were similar in the majority of studies (Supplementary Table S5). The most commonly used radiolabelled FAPI molecule was [^68^Ga]Ga-FAPI-04 in 20 studies (71%), followed by [^68^Ga]Ga-FAPI-46, which was used in six studies (21%). The injected dose ranged from 1.8 to 3.7 MBq/kg for radiolabelled FAPI and from 2.59 to 5.55 MBq/kg for [^18^F]FDG. Three studies used PET/MRI scanners: Zhang et al. used PET/MRI in all patients, Siripongsatian et al. performed both PET/CT and PET/MRI scans, while Hirmas et al. used PET/MRI only in 7 of 324 patients (2%) [30,48,52]. All other studies were performed using PET/CT scanners, using static protocols. Two studies performed multiple time-point imaging (at 60 and 180 min, and at 10, 60 and 180 min) [32,35]. Time-of-flight reconstruction algorithms were used in four studies [42,46,47,48]. Twenty-one studies (75%) scanned 60 min after administration of the tracer and the study by Hirmas et al. scanned at the earliest time-point with a median time of 14 min (interquartile range (IQR) 24 min) after tracer administration [30]. When both FAPI and [^18^F]FDG PET imaging were performed, a maximum interval of seven days between the scans was applied. In all studies, the interpretation of the scans was performed by two or more medical specialists, either nuclear medicine physicians or radiologists. In ten studies, interpretations were conducted independently from each other. However, in two studies, the methodology of interpretation was not reported [14,30]. The availability of clinical or imaging data during the interpretation varied, with 17 studies lacking blinding. In 26 studies, the primary tumour was confirmed by histopathology. The most commonly used reference standard for metastases was imaging or imaging in combination with another modality (Table 1).

### 3.3. Patient Analysis

**Figure 3:**
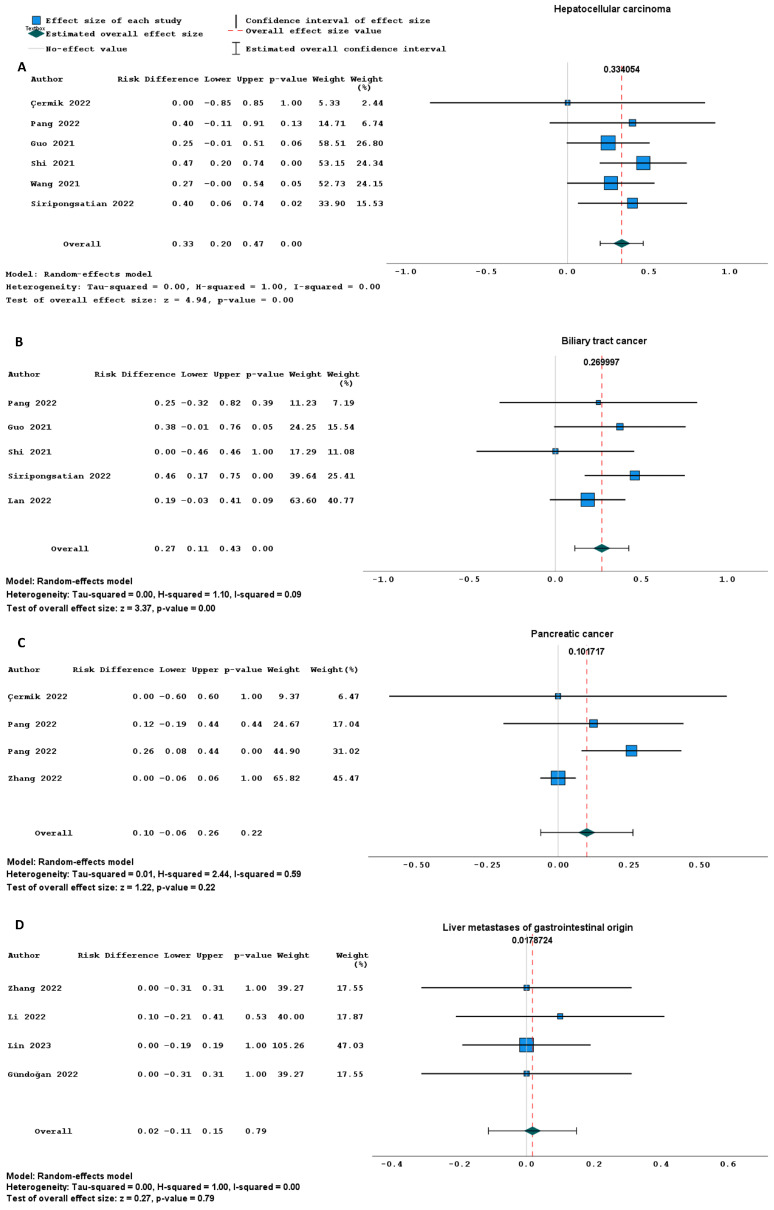
(**A**–**D**) Forest plots comparing the patient-based detection rate of radiolabelled FAPI versus [^18^F]FDG PET imaging. These plots present a patient-based analysis of the detection rates of radiolabelled FAPI compared to [^18^F]FDG PET imaging for hepatocellular carcinoma (**A**), biliary tract cancer (**B**), pancreatic cancer (**C**) and liver metastases of gastrointestinal origin (**D**) expressed as a risk difference. For each cancer type, the plot shows the estimated effect size and its corresponding confidence interval for each study. At the bottom, the overall effect size with its confidence interval is provided [26,29,32,38,41,42,43,44,46,48,49,52]. Abbreviations: FAPI: fibroblast activation protein inhibitor, [^18^F]FDG: 2-deoxy-2-[^18^F]fluoro-D-glucose, PET: positron emission tomography.

In the patient-based analysis, the detection rate for HCC and BTC was significantly higher for FAPI PET imaging, compared to [^18^F]FDG PET (risk difference 0.33, 95% CI: 0.20–0.47, I^2^: 0% and 0.27, 95% CI: 0.11–0.43, I^2^: 9%, respectively) (Figure 3A,B). In PC and liver metastases of gastrointestinal origin, no significant difference was observed (Figure 3C,D). For the analysis of extra-hepatic distant and lymph node metastases, insufficient data were available.

### 3.4. Lesion Analysis

In the lesion-based analysis, the detection rate of FAPI PET imaging for primary tumours was also significantly higher in HCC and BTC than for [^18^F]FDG PET (risk difference 0.34, 95% CI: 0.23–0.45, I^2^: 18% and 0.28, 95% CI: 0.08–0.48, I^2^: 61%, respectively). In PC, no difference was observed (risk difference 0.09, 95% CI: −0.06–0.24, I^2^: 50%). Radiolabelled FAPI outperformed [^18^F]FDG in the detection of lymph node, liver and extra-hepatic distant metastases (Supplementary Figure S1A–C). The complete data sets and analyses are presented in Supplementary Tables S6–S8.

### 3.5. Semi-Quantitative Analysis

**Figure 4:**
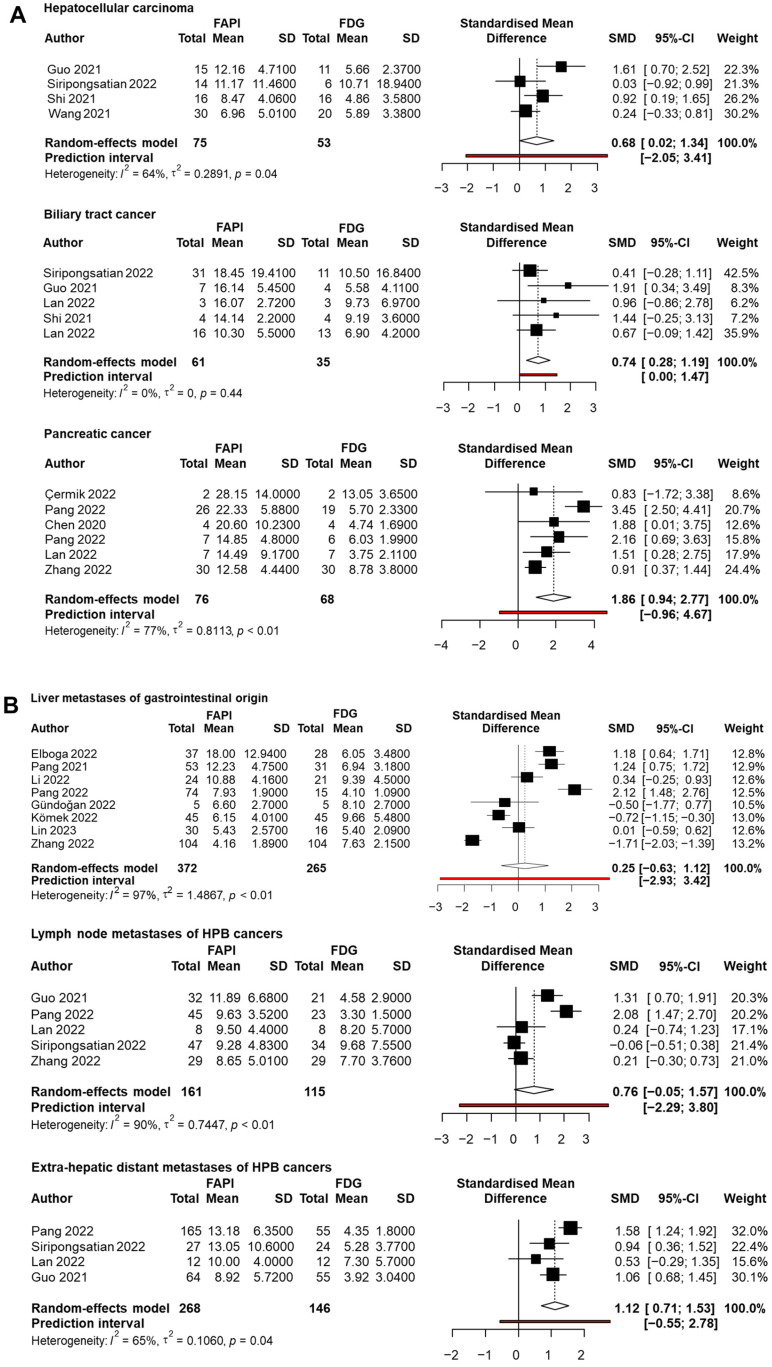
(**A**,**B**). Forest plots comparing the mean SUV max of radiolabelled FAPI versus [^18^F]FDG PET imaging. These plots show a direct comparison of mean SUVmax of radiolabelled FAPI versus [^18^F]FDG PET imaging in primary hepato-pancreato-biliary cancers (**A**) and metastases (**B**), expressed as a standardised mean difference. For each category, the plot shows the estimated effect size and its corresponding confidence interval for each study. The overall effect size with its confidence interval is provided at the bottom of the plot [26,27,29,31,32,37,38,39,40,41,42,43,44,46,48,49,52]. Abbreviations: SUVmax: maximum standardised uptake values, FAPI: fibroblast activation protein inhibitor, FDG: 2-deoxy-2-[^18^F]fluoro-D-glucose, PET: positron emission tomography, SD: standard deviation, SMD: standardised mean difference, CI: confidence interval.

The tracer uptake of radiolabelled FAPI was highest in PC, with a mean SUVmax of 15.64 (95% CI: 12.41–18.87, I^2^: 88%), followed by BTC 13.47 (95% CI: 11.60–15.33, I^2^: 58%), extra-hepatic distant metastases, lymph node metastases, liver metastases of gastrointestinal origin and HCC, respectively (Table 2). In a head-to-head comparison of the mean SUVmax, the radiolabelled FAPI uptake was significantly higher in all primary tumours and extra-hepatic distant metastases. In lymph node metastases and liver metastases of gastrointestinal origin, no significant difference in tracer uptake was found (Figure 4A,B). The TBR was rarely reported, with sufficient data for analysis only available for HCC, BTC and liver metastases of gastrointestinal origin. All studies used a region-of-interest in normal liver tissue as the background comparison. In these subgroups, the TBR was considerably higher with radiolabelled FAPI PET imaging, compared to [^18^F]FDG PET (Table 2). The complete data sets of SUVmax and TBR are available in Supplementary Tables S9 and S10.

## 4. Discussion

This systematic review and meta-analysis investigating the diagnostic performance of radiolabelled FAPI PET imaging in comparison to [^18^F]FDG in HPB malignancies, including 28 studies comprising 1621 patients, showed a higher detection rate of FAPI PET imaging in HCC and BTC, in the patient- and lesion-based analyses. In PC, the detection rate between the tracers did not differ in either analysis. The lesion-based analyses revealed significantly higher detection rates for metastases with FAPI PET imaging. All HPB malignancies showed a higher tracer uptake of radiolabelled FAPI compared to [^18^F]FDG.

The heterogeneity of the study results varied. The heterogeneity of the patient-based detection rate was low, with the exception of PC. In the lesion-based results, it was moderate to high, while the semi-quantitative metrics showed the highest rate of heterogeneity. Despite similar designs, there were relevant differences among the studies in terms of the availability of clinical and imaging data during the interpretation of the scan, the histopathological confirmation of metastases and the patient characteristics.

The high detection rate and tracer uptake of radiolabelled FAPI compared to [^18^F]FDG PET imaging in HPB malignancies has multiple contributing factors. First, the high physiological demand of glucose and therefore high uptake of [^18^F]FDG in the gastrointestinal tract and liver creates a higher background signal in [^18^F]FDG PET imaging. Likewise, the obstruction of the biliary or pancreatic duct by the tumour causes inflammation which may mimic the [^18^F]FDG uptake in the tumour, and hamper the interpretation. Second, the strong fibrotic reaction of HPB malignancies with CAFs recruitment and concomitant FAP expression provide abundant target expression for potential binding of radiolabelled FAPI. Lastly, the low physiological expression of FAP in the gastrointestinal tract and high specificity of the FAPI tracer for the target facilitate an improved tumour-to-background distinction.

This is the first systematic review and meta-analysis to separately determine the detection rate and semi-quantitative metrics of FAPI PET imaging in HPB malignancies in comparison to [^18^F]FDG. Previous meta-analyses of FAPI PET imaging have investigated the detection rate of gastrointestinal tumours or a variety of oncological diseases [53,54]. Huang et al. performed a subgroup analysis of the detection rate of FAPI PET in pancreatic cancer (1.00, 95% CI: 0.22–1.00, I^2^: 94%), but they did not compare these results with those of [^18^F]FDG PET imaging, which is considered the standard comparator, and their results exhibited a high degree of heterogeneity. Additionally, a systematic review by Veldhuijzen van Zanten and colleagues observed a higher detection rate of radiolabelled FAPI versus [^18^F]FDG PET imaging for pancreatic- and cholangiocarcinoma [55]. However, they did not conduct statistical analyses on the reported detection rates of the included studies or include studies on HCC in their review.

The main limitation of this meta-analysis is the presence of a high degree of heterogeneity in the study results. A probable cause is the presence of a selection bias, due to variations in clinical setting, disease stage or prior treatments. Also, diversity in study acquisition protocols and sample size may have influenced the consistency of the results. Technical aspects may have introduced heterogeneity in the semi-quantitative metrics through the use of different types of scanners, types of FAPI tracers and image reconstruction and interpretation methods. Unfortunately, the number of studies per tumour type or clinical setting was too low to address these limitations through subgroup or meta-regression analysis. Additionally, the presence of verification bias in metastases limits the interpretation of the results. Not all suspected metastases were histopathologically confirmed or sufficiently followed-up with imaging due to ethical reasons. Furthermore, several research groups conducted studies in which it was not clearly documented whether there was a potential overlap among the patients who participated. Finally, it was not possible to address the potential bias from the lack of blinding during the interpretation of the scans.

Our findings are in line with the previous results of systematic reviews of FAPI PET imaging in gastrointestinal malignancies [53,54,55]. Importantly, our meta-analysis shows that FAPI PET imaging seems to have benefits in HPB cancer diagnostics in comparison to [^18^F]FDG PET and may assist clinicians in case of a diagnostic dilemma. The more homogenous results of our analyses, particularly in the patient-based analysis, along with the diagnostic challenge posed by HPB tumours and the larger number of included patients for each tumour type, distinguishes this meta-analysis from the previously published reviews and meta-analyses. In the future, FAPI PET imaging may improve surgical patient selection, by detecting small metastases before surgery or by using the uptake as an imaging biomarker to risk-stratify patients. An example of risk stratifying has recently been published by Strating et al., in which they identified patients with CMS-4 subtype metastases of colorectal cancer with FAPI imaging [56].

Future research on FAPI PET imaging in HPB malignancies should focus on the potential clinical impact of FAPI imaging, in patients with resectable disease to prevent unnecessary surgery, or on the application of FAPI as a theranostic modality. Additionally, larger sample sizes, more histopathological confirmation or sufficient imaging follow-up of metastases would strengthen the quality of the studies.

## 5. Conclusions

In conclusion, FAPI PET imaging seems to be beneficial in comparison to [^18^F]FDG PET in HPB cancer diagnostics. The detection rate and tracer uptake of radiolabelled FAPI was higher than [^18^F]FDG in HCC and BTC. In PC, the tracer uptake of radiolabelled FAPI was higher, yet the detection rate did not differ. Improved surgical patient selection and stratification of high-risk patients may provide the greatest added value of FAPI PET imaging. Larger, head-to-head diagnostic accuracy studies for specific HPB tumour types are warranted.

## Figures and Tables

**Table 1:** Study characteristics.

Authors	Year	Country	Design	Scope	N	Patients Per Subgroup	Radiotracer (Number of Patients if Stated)	Injected Activity (Mean or Otherwise Specified)	Reference Standard (Number of Patients if Stated)	Imaging Modality Used as Comparison to FAPI PET/CT (Maximum Interval in Days or Otherwise Specified)
Çermik [26]	2022	Turkey	P	General	42	2 PC, 1 HCC	[^68^Ga]Ga-FAPI-04	FAPI: 1.85 MBq/kg [^18^F]FDG: 3.7 MBq/kg	Combination of methods	[^18^F]FDG PET/CT (7d)
Chen [27] *	2020	China	P	General	75	4 PC, 6 HCC, 5 CCA	[^68^Ga]Ga-FAPI-04	FAPI: 1.8–2.2 MBq/kg [^18^F]FDG: 3.7 MBq/kg	Prim: HP Meta: HP	[^18^F]FDG PET/CT (7d)
Chen [36]	2021	China	P	General	73	6 LC, 1 CCA, 1 PC	[^68^Ga]Ga-FAPI-04	FAPI: 1.8–2.2 MBq/kg [^18^F]FDG: 3.7 MBq/kg	HP (49) imaging (19)	[^18^F]FDG PET/CT (7d)
Dendl [28] †	2022	Germany	R	General	55	5 CCA, 2 HCC	[^68^Ga]Ga-FAPI-04 (4) [^68^Ga]Ga-FAPI-46 (3)	FAPI: median 252 MBq	NR	No comparison
Elboga [37]	2022	Turkey	R	GIS	37	10 PBC, 37 LM (Lesions)	[^68^Ga]Ga-FAPI-04	FAPI: 2 MBq/kg [^18^F]FDG: 370–555 MBq	Prim: HP Meta: combination	[^18^F]FDG PET/CT (mean 3d)
Gündoǧan [38]	2022	Turkey	P	Gastric	21	5 LM	[^68^Ga]Ga -FAPI-04	FAPI: 2 MBq/kg [^18^F]FDG 3.5–5.5 MBq/kg	Prim: HP Meta: imaging	[^18^F]FDG PET/CT (7d)
Guo [29] *	2021	China	R	Liver	34	20 HCC, 12 CCA	[^68^Ga]Ga-FAPI-04	FAPI: 148–259 MBq [^18^F]FDG: 3.7 MBq/kg	HP, otherwise clinical or imaging FU	Imaging (ce-CT, MRI and [^18^F]FDG PET/CT) (7d)
Hirmas [30] †	2022	Germany	P	General	324	67 PC, 11 CCA	[^68^Ga]Ga-FAPI-04 (21); [^68^Ga]Ga-FAPI-46 (303)	FAPI: median 112 MBq [^18^F]FDG: median 283 MBq	HP, Meta: NR	[^18^F]FDG PET/CT (median 0d, IQR 2)
Koerber [34]	2020	Germany	R	CRC	22	14 LM	[^68^Ga]Ga-FAPI-04 (16); [^68^Ga]Ga FAPI-46 (6)	FAPI: 111–298 MBq	Imaging	conventional imaging (NR)
Kömek [39]	2022	Turkey	P	CRC	39	7 LM	[^68^Ga]Ga FAPI-04	FAPI: 2 MBq/kg [^18^F]FDG: 3.5–5.5 MBq/kg	HP	[^18^F]FDG PET/CT (7d)
Kratochwil [14]	2019	Germany	R	General	80	51 PC, 5 HCC, 12 CCA	[^68^Ga]Ga-FAPI-04	FAPI: 122–312 MBq	Prim: HP, Meta: HP or imaging	No comparison
Lan [41]	2022	China	P	BTC	19	9 CCA, 9 GB	[^68^Ga]Ga-FAPI-04	FAPI: 1.85 MBq/kg [^18^F]FDG 3.7 MBq/kg	Prim: HP, Meta: HP or imaging	[^18^F]FDG PET/CT (3d)
Lan [40]	2022	China	P	General	123	16 LC, 7 PC, 1 CCA, 2 GB	[^68^Ga]Ga-FAPI-04	FAPI: 1.85 MBq/kg [^18^F]FDG 3.7 MBq/kg	Prim: HP, Meta: HP or imaging	[^18^F]FDG PET/CT (3d)
Li [42]	2022	China	R	GIS	51	10 LM	[^68^Ga]Ga-FAPI-04	FAPI 1.85–3.70 MBq/kg, [^18^F]FDG 3.7–5.55 MBq/kg	Prim: HP Meta: HP or imaging FU	[^18^F]FDG PET/CT (median 1d, IQR: 1–1)
Lin [43]	2023	China	P	CRC	61	9 LM	[^68^Ga]Ga-FAPI-04	FAPI 1.85–2.96 MBq/kg	Prim: HP Meta: HP (10), imaging (24)	[^18^F]FDG PET/CT (7d)
Pang [31] *	2021	China	R	GIS	35	10 LM	[^68^Ga]Ga-FAPI-04	FAPI: 1.8–2.2 MBq/kg [^18^F]FDG: 3.7 MBq/kg	HP (primary and metastases)	[^18^F]FDG PET/CT (median 2d, range 1–6d)
Pang [32] *	2022	China	R	Pancreas	36	26 PC	[^68^Ga]Ga-FAPI-04	FAPI: 1.8–2.2 MBq/kg [^18^F]FDG: 3.7 MBq/kg	Prim: HP, Meta: HP or imaging FU	[^18^F]FDG PET/CT (1–6d)
Pang [44]	2022	China	P	General	64	5 HCC, 3 CCA, 7 PC	[^68^Ga]Ga-FAPI-2286 [^68^Ga]Ga-FAPI-46	FAPI-46: 194 MBq FAPI-2286: 195 MBq [^18^F]FDG: 288 MBq	Prim and meta: HP or imaging	[^18^F]FDG PET/CT (7d)
Röhrich [35]	2021	Germany	R	Pancreas	19	19 PC	[^68^Ga]Ga-FAPI-04 (16); [^68^Ga]Ga-FAPI-46 (3)	FAPI: 167–293 MBq	Prim: HP Meta: imaging	ce-CT (mean 17.6d)
Şahin [45]	2021	Turkey	R	GIS	31	9 PC, 31 LM	[^68^Ga]Ga-FAPI-04	FAPI: 2–3 MBq/kg [^18^F]FDG: 5 MBq/kg	HP (5), imaging and clinical (26)	[^18^F]FDG PET/CT (minimum 2 week interval)
Shi [47]	2021	China	P	Liver	17	11 HCC, 2 CCA, 3 LM	[^68^Ga]Ga-FAPI-04	FAPI: 96–260 MBq	HP	Conventional imaging (NR)
Shi [46]	2021	China	P	Liver	20	14 HCC, 3 CCA	[^68^Ga]Ga-FAPI-04	FAPI: 196–260 MBq [^18^F]FDG: 3.7 MBq/kg	HP (15), imaging (5)	[^18^F]FDG PET/CT (3d)
Siripongsatian [48]	2022	Thailand	R	Liver	27	14 HCC, 13 CCA	[^68^Ga]Ga-FAPI-46	FAPI and [^18^F]FDG: 2.59 MBq/kg	HP and MRI imaging	[^18^F]FDG PET/CT (7d)
Wang [49]	2021	China	R	Liver	29	26 HCC	[^68^Ga]Ga-FAPI-04	FAPI: 185 MBq [^18^F]FDG: NR	HP (24) imaging (5)	[^18^F]FDG PET/CT (1d)
Wu [50]	2022	China	R	GIS	35	48 LM (lesions)	[^18^F]-FAPI-42	FAPI: 259 MBq [^18^F]FDG: 5.18 MBq/kg	HP (26), imaging (9)	[^18^F]FDG PET/CT (7d)
Zhang [52]	2022	China	P	Pancreas	33	30 PC	[^68^Ga]Ga-FAPI-04	FAPI: 1.85–3.70 MBq/kg [^18^F]FDG: 3.70–5.55 MBq/kg	Prim: HP, Meta: imaging FU	FAPI PET/MRI versus [^18^F]FDG PET/CT (median 2d)
Zhang [51]	2022	China	P	Liver	37	20 HCC, 3 CCA, 2 LM	[^18^F]-FAPI	FAPI: 148–259 MBq [^18^F]FDG: 3.7 MBq/kg	HP (34), imaging and clinical FU (3)	[^18^F]FDG PET/CT (7d)
Zheng [33] *	2021	China	R	General	182	6 PC, 4 HCC, 2 CCA, 1 GB	[^68^Ga]Ga-FAPI-04	FAPI: 3.7 MBq/kg	NR	No comparison

Abbreviations: * common study registration, †: part of previous publication, P: prospective, R: retrospective, GIS: gastrointestinal, CRC: colorectal carcinoma, BTC: biliary tract cancer, PC: pancreatic cancer, PBC: pancreatobiliary cancer, LC: liver cancer, HCC: hepatocellular carcinoma, CCA: cholangiocarcinoma, GB: gallbladder carcinoma, BTC: biliary tract cancer, LM: liver metastases of gastrointestinal origin, IQR: interquartile range, NR: not reported, FAPI: fibroblast activation protein inhibitor, [^18^F]FDG: 2-deoxy-2-[^18^F]fluoro-D-glucose, Prim: primary tumour, Meta: metastases, HP: histopathology, FU: follow-up, MRI: magnetic resonance imaging, PET/CT: positron emission tomography/computed tomography, ce-CT: contrast-enhanced computed tomography.

**Table 2:** Meta-analyses of the semi-quantitative metrics of the primary hepato-pancreato-biliary tumours and metastases.

		HCC	BTC	PC	Liver Metastases *	LN Metastases	Extra-Hepatic Distant Metastases
**FAPI SUV max**	Mean	7.9 (5.49–10.31)	13.47 (11.60–15.33)	15.64 (12.41–18.87)	8.64 (6.35–10.93)	9.64 (8.95–10.34)	10.32 (8.01–12.63)
	I^2^	95%	58%	88%	97%	25%	94%
**[^18^F]** **FDG SUV max**	Mean	5.56 (4.68–6.44)	8.07 (5.95–10.19)	6.46 (4.43–8.50)	7.04 (5.68–8.39)	6.43 (4.03–8.84)	4.35 (3.96–4.75)
	I^2^	0%	28%	85%	95%	93%	48%
**FAPI TBR**	Mean	7.62 (5.46–9.78)	17.19 (8.02–26.36)		6.08 (3.29–8.87)		
	I^2^	77%	96%		88%		
**[^18^F]** **FDG TBR**	Mean	2.49 (1.71–3.26)	2.34 (0.86–3.81)		3.31 (1.89–4.74)		
	I^2^	60%	0%		90%		

Abbreviations: FAPI: fibroblast activation protein inhibitor, [^18^F]FDG: 2-deoxy-2-[^18^F]fluoro-D-glucose, SUV max: maximum standardised uptake value, TBR: tumour to background ratio, HCC: hepatocellular carcinoma, BTC: biliary tract cancer, PC: pancreatic cancer, LN: lymph node, *: liver metastases of gastrointestinal origin. In brackets, the 95% confidence interval is shown.

## Data Availability

All data generated or analysed during this study are included in the article and the Supplementary Materials.

## Supplementary Materials

The supporting information can be downloaded at: https://www.mdpi.com/article/10.3390/ijms26051978/s1. Reference [57] is cited in the Supplementary Materials.
