# Supplementary material for: Diagnostic Performance of Radiolabelled FAPI Versus [18F]FDG PET Imaging in Hepato-Pancreato-Biliary Oncology: A Systematic Review and Meta-Analysis
Source: Int J Mol Sci. 2025 Feb 25;26(5):1978. doi: 10.3390/ijms26051978 (PMC11900289; doi:10.3390/ijms26051978)
Supplement: Supplementary file 1 [file ijms-26-01978-s001.zip › Supplemental File_Systematic review FAPI HPB_25022025.pdf]

Supplementary materials

Diagnostic Performance of Radiolabelled FAPI Versus [^{18}F]FDG PET Imaging in Hepato-Pancreato-Biliary Oncology: A Systematic Review and Meta-Analysis

Supplemental Table S1

Search strategy in PubMed

Search	Query	Results
#3	#1 AND #2	2,202
#2	FAP[tiab] OR FAPI[tiab] OR "fibroblast activation protein*"[tiab]	6,799
#1	"Pancreatic Neoplasms"[Mesh] OR "Cholangiocarcinoma"[Mesh] OR "Carcinoma, Hepatocellular"[Mesh] OR ((pancrea*[tiab] OR liver*[tiab] OR gallbladder*[tiab] OR "gall bladder*"[tiab] OR HPB[tiab] OR "Hepato-pancreato-biliary"[tiab] OR Hepatocellular*[tiab] OR "Hepato cellular*"[tiab] OR bileduct*[tiab] OR "bile duct*"[tiab] OR colorectal*[tiab] OR colon*[tiab] OR rect*[tiab] OR bowel*[tiab]) AND (Neoplasm*[tiab] OR cancer*[tiab] OR tumor*[tiab] OR tumour*[tiab] OR carcinoma*[tiab] OR adenocarcinoma*[tiab])) OR Cholangiocarcinoma*[tiab]	767,115

Supplemental Table S2

Search strategy in Embase.com

Search	Query	Results
**#5**	#4 NOT OLD PUIs	**33**
**#4**	#3 AND ('article'/it OR 'article in press'/it OR 'review'/it)	**2,372**
**#3**	#1 AND #2	**3,714**
**#2**	(FAP OR FAPI OR ‘fibroblast activation protein*’):ti,ab,kw	**10,744**
**#1**	'pancreas tumor'/exp OR 'biliary tract cancer'/exp OR 'hepatobiliary system cancer'/exp OR ((pancrea* OR liver* OR gallbladder* OR ‘gall bladder*’ OR HPB OR ‘Hepato-pancreato-biliary’ OR Hepatocellular* OR ‘Hepato cellular*’ OR bileduct* OR ‘bile duct*’ OR colorectal* OR colon* OR rect* OR bowel*):ti,ab,kw AND (Neoplasm* OR cancer* OR tumor* OR tumour* OR carcinoma* OR adenocarcinoma*):ti,ab,kw) OR Cholangiocarcinoma*:ti,ab,kw	**1,197,022**

Supplemental Table S3

Search strategy in Web of Science

Search	Query	Results
**#3**	#1 AND #2	**2,537**
**#2**	TS=(FAP OR FAPI OR “fibroblast activation protein*”)	**8,920**
**#1**	TS=(((pancrea* OR liver* OR gallbladder* OR “gall bladder*” OR HPB OR “Hepato-pancreato-biliary” OR Hepatocellular* OR “Hepato cellular*” OR bileduct* OR “bile duct*” OR colorectal* OR colon* OR rect* OR bowel*) AND (Neoplasm* OR cancer* OR tumor* OR tumour* OR carcinoma* OR adenocarcinoma*)) OR Cholangiocarcinoma*)	**937,091**

Supplemental Table S4

Search strategy in Cochrane

Search	Query	Results
**#3**	#1 AND #2	**95**
**#2**	(FAP OR FAPI OR Fibroblast NEXT activation NEXT protein*):ti,ab,kw	**410**
**#1**	(((pancrea* OR liver* OR gallbladder* OR gall NEXT bladder* OR HPB OR Hepato NEXT pancreato NEXT biliary OR Hepatocellular* OR Hepato NEXT cellular* OR bileduct* OR bile NEXT duct* OR colorectal* OR colon* OR rect* OR bowel*) AND (Neoplasm* OR cancer* OR tumor* OR tumour* OR carcinoma* OR adenocarcinoma*)) OR Cholangiocarcinoma*):ti,ab,kw	**56,551**

Supplementary Table S5. Additional clinical characteristics of included studies.

Author	Year	Scope	Clinical setting	N	Patients per subgroup	Age (In years)	Sex (Female/ Male)	Image acquisition protocol
**Çermik [26]**	2022	General	(re)staging	42	2 PC, 1 HCC	Mean 58.5 (31-84 range)	16/26	NR
**Chen [27] ***	2020	General	(re)staging	75	4 PC, 6 HCC, 5 CCA	Median 61,5 (range 32-85)	28/47	Whole-Body, 6-8 BP 2-2.5 min/BP
**Chen [36]**	2021	General	Inconclusive FDG PET findings	73	6 LC, 1 CCA, 1 PC	Median 57 (range 24-85)	28/40	Whole-Body, 6-8 BP 2-2.5 min/BP
**Dendl [28] †**	2022	General	Rare malignancy with diagnostic challenge	55	5 CCA, 2 HCC	Median: 60 (range/IQR NR)	14/41	Whole-body, time per BP NR
**Elboga [37]**	2022	GIS	Metastatic disease (peritoneal)	37	10 PBC, 37 LM (Lesions)	Mean 62.8 (SD 12.7)	14/23	Whole-body, 2.5 min/BP
**Gündoğan [38]**	2022	Gastric	(re)staging	21	5 LM	Median 61 (40-81)	9/12	Whole-body, 2.5 min/BP
**Guo [29] ***	2021	Liver	Doubtful lesions or (re)staging	34	20 HCC, 12 CCA	Mean 60.6 (33-75 range)	9/25	Whole-body, 6-8 BP, 2-2.5 min/BP
**Hirmas [30]†**	2022	General	Proven or suspected malignancy	324	67 PC, 11 CCA	Median 59 (IQR 16)	156/168	Whole-body, 3-5 min/BP
**Koerber [34]**	2020	CRC	Diagnostic challenge	22	14 LM	Median 62 (range 38-79)	12/10	Whole-body, time per BP NR
**Kömek [39]**	2022	CRC	(re)staging	39	7 LM	Median 61 (range 29-83)	17/22	Whole-body, 2.5 min/BP
**Kratochwil [14]**	2019	General	Diagnostic challenge	80	51 PC, 5 HCC, 12 CCA	NR	NR	Whole-body, time per BP NR
**Lan [41]**	2022	BTC	(re)staging	19	9 CCA, 9 GB	Mean 61 (SD 10)	12/6	Whole-body, 3min/BD
**Lan [40]**	2022	General	(re)staging	123	16 LC, 7 PC, 1 CCA, 2 GB	Mean 56.11 (SD 11.94)	54/69	Whole-body, 5-6 BP, 3 min/BP
**Li [42]**	2022	GIS	(re)staging	51	10 LM	Median 57 (IQR 48-66)	20/31	Whole-body, 7-8 BP, time per BP NR
**Lin [43]**	2023	CRC	(re)staging to aid clinical decision making	61	9 LM	Median 62 (range 32-81)	19/42	Whole-body, 6-8 BP, 1 min/BP
**Pang [31] ***	2021	GIS	(re)staging	35	10 LM	Median 64 (IQR 53-68)	17/18	NR
**Pang [32]***	2022	Pancreas	Discriminating mass lesion or staging	36	26 PC	Median 60 (IQR 48-71)	11/25	Whole-body, 2-3 BP, 2.5 min/BP
**Pang [44]**	2022	General	(re)staging	64	5 HCC, 3 CCA, 7 PC	Median 57.5 (range 32-85)	26/38	NR
**Röhrich [35]**	2021	Pancreas	Aid clinical decision making	19	19 PC	Median 64 (range 52-80)	9/10	Whole-body, time per BP NR
**Şahin [45]**	2021	GIS	(re)staging	31	9 PC, 31 LM	Mean 61.9 (SD 10.9)	12/19	NR

**Shi [47]**	2021	Liver	Suspected malignancy	17	11 HCC, 2 CCA, 3 LM	Mean 62.9 (SD 8.0)	4/13	NR
**Shi [46]**	2021	Liver	Suspected malignancy	20	14 HCC, 3 CCA	Mean 58 (SD 9.4)	2/18	Whole-body, 2 min/BP
**Siripongsatian [48]**	2022	Liver	Diagnosed or suspected malignancy	27	14 HCC, 13 CCA	Median 68 (IQR 60-74)	6/21	Whole-body, continuous bed motion 1.6-1.8 mm/s
**Wang [49]**	2021	Liver	Suspected or recurrent malignancy	29	26 HCC	Mean 59.4 (SD 6.9)	1/24	Whole-body, time per BP NR
**Wu [50]**	2022	GIS	Recurrent or metastatic disease	35	48 LM (lesions)	Median 54 (range 32 - 76)	14/21	Whole-body, time per BP NR
**Zhang [52]**	2022	Pancreas	Suspected malignancy	33	30 PC	Mean 66.9 (range 48-81)	14/19	Whole-body, 5-6 BP, 3 min/BP
**Zhang [51]**	2022	Liver	Suspected malignancy , inconclusive FDG result	37	20 HCC, 3 CCA, 2 LM	Median 57 (range 48-67)	3/34	NR
**Zheng [33] ***	2021	General	(re)staging	182	6 PC, 4 HCC, 2 CCA, 1 GB	Median 57 (range 20-87)	70/112	Whole-body, 6-8 BP, 3 min/BP

Abbreviations: *: common study registration, † : part of previous publication, GI: gastrointestinal, CRC: colorectal carcinoma, BTC: biliary tract cancer, CUP: carcinoma of unknown primary, PC: pancreatic cancer, PBC: pancreatobiliary cancer, LC: liver cancer, HCC: hepatocellular carcinoma, CCA: cholangiocarcinoma, GB: gallbladder carcinoma, BTC: biliary tract cancer, LM: liver metastases of gastrointestinal origin, SD: standard deviation, IQR: interquartile range, NR: not reported, BP: bed position

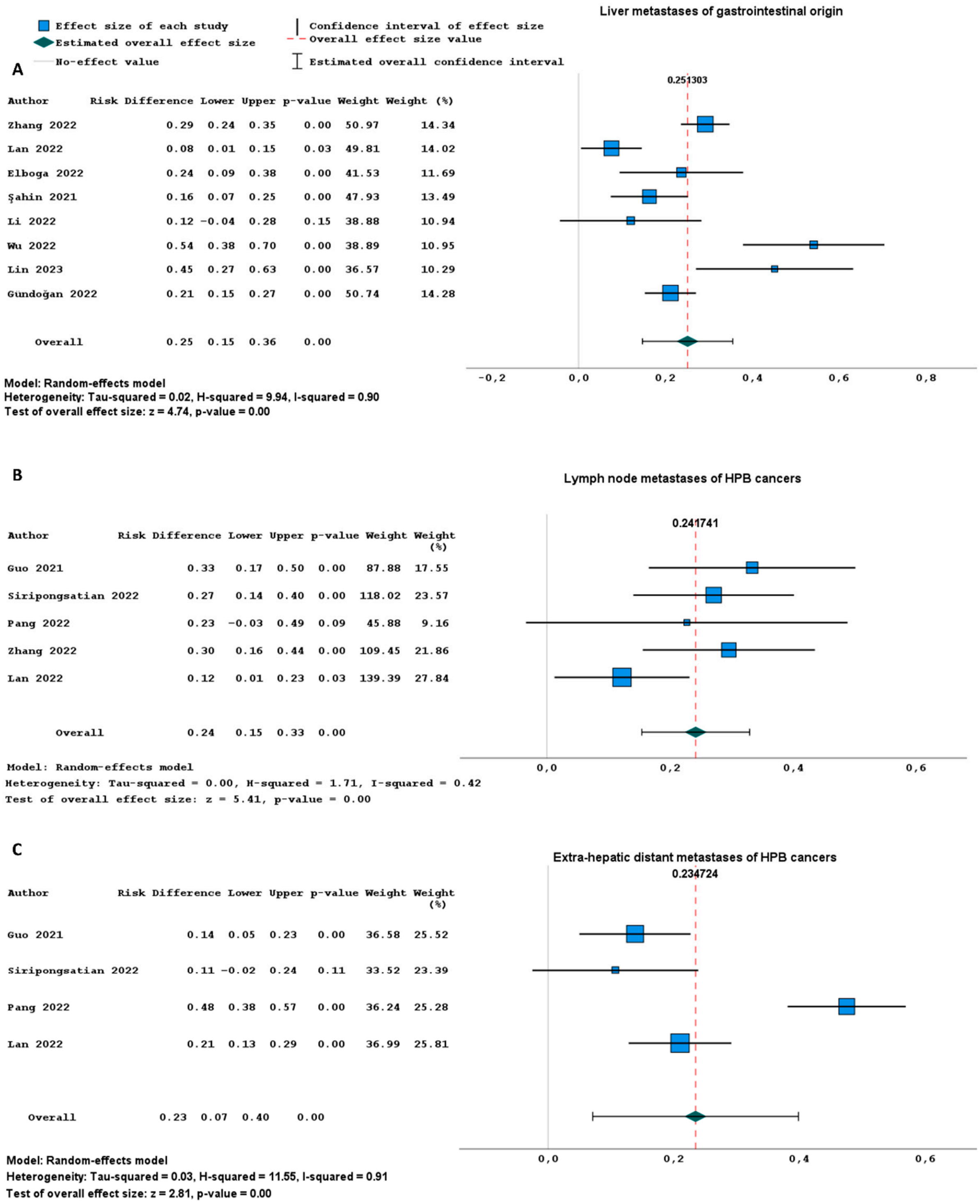

Supplemental Figure S1. (A-C). Forest plots comparing the lesion-based detection rate of radiolabelled FAPI versus [^{18}F]FDG PET imaging. These forest plots present a lesion-based analysis of the detection rates of radiolabelled FAPI compared to [^{18}F]FDG PET imaging for liver metastases of gastrointestinal origin (A), lymph node metastases of hepato-pancreato-biliary (HPB) cancers (B) and extra-hepatic distant metastases of HPB cancers (C) expressed as a risk difference. For each cancer type, the plot shows the estimated effect size and its corresponding confidence interval for each study. At the bottom, the overall effect size with its confidence interval is provided [52,41,37,45,42,50,43,38,29,48,32].

Abbreviations: FAPI: fibroblast activation protein inhibitor, [^{18}F]FDG: 2-deoxy-2-[^{18}F]fluoro-D-glucose, PET: positron emission tomography

Supplemental Table S6. Complete data of the positive detection rate analysis (risk difference) between radiolabelled FAPI and [18F]FDG PET imaging for primary HPB tumours.

**Hepatocellular carcinoma**	**Patient-based analysis**						**Lesion-based analysis**					
	**Number of patients**	**FAPI PET**		**[18F]FDG PET**		**Risk Difference (95% CI)**	**Number of lesions**	**FAPI PET**		**[18F]FDG PET**		**Risk Difference (95% CI)**
**Author**		**positive event**	**negative event**	**positive event**	**negative event**			**positive event**	**negative event**	**positive event**	**negative event**	
Çermik (26)	1	1.5	0.5	1.5	0.5	0.00 (−0.85 − 0.85)						
Chen (27)							6	6.5	0.5	5.5	1.5	0.14 (−0.22 – 0.50)
Lan (40)							16	16.5	0.5	12.5	4.5	0.24 (0.01 – 0.46)
Pang (44)	4	4.5	0.5	2.5	2.5	0.40 (−0.11 – 0.91)	4	4.5	0.5	2.5	2.5	0.40 (−0.11 – 0.91)
Guo (29)	16	15	1	11	5	0.25 (−0.01 – 0.51)	16	15	1	11	5	0.25 (−0.01 – 0.51)
Shi (46)	14	14.5	0.5	7.5	7.5	0.47 (0.20 – 0.74)	16	16.5	0.5	7.5	9.5	0.53 (0.28 – 0.78)
Wang (49)	15	14	1	10	5	0.27 (0.00 – 0.54)	35	30	5	20	15	0.29 (0.08 – 0.49)
Siripongsatian (48)	9	9.5	0.5	5.5	4.5	0.40 (0.06 – 0.74)	14	14.5	0.5	6.5	8.5	0.53 (0.27 – 0.80)
Shi (47)	11	11.5	0.5				15	15.5	0.5			
Zhang (51)	25	24	1				33	32	1			
**Overall**						**0.33 (0.20 – 0.47)**						**0.34 (0.23 − 0.45)**

**Biliary tract cancer**	**Patient-based analysis**						**Lesion-based analysis**					
	**Number of patients**	**FAPI PET**		**[18F]FDG PET**		**Risk Difference (95% CI)**	**Number of lesions**	**FAPI PET**		**[18F]FDG PET**		**Risk Difference (95% CI)**
**Author**		**positive event**	**negative event**	**positive event**	**negative event**			**positive event**	**negative event**	**positive event**	**negative event**	
Chen (27)							5	5.5	0.5	3.5	2.5	0.33 (−0.12 – 0.79)
Lan (40)							3	3.5	0.5	3.5	0.5	0.00 (−0.46 – 0.46)
Pang (44)	3	3.5	0.5	2.5	1.5	0.25 (−0.32 – 0.82)	3	3.5	0.5	2.5	1.5	0.25 (−0.32 – 0.82)
Guo (29)	7	7.5	0.5	4.5	3.5	0.38 (−0.01 – 0.76)	7	7.5	0.5	4.5	3.5	0.38 (−0.01 – 0.76)
Shi (46)	3	3.5	0.5	3.5	0.5	0.00 (−0.46 – 0.46)	4	4.5	0.5	4.5	0.5	0.00 (−0.37 – 0.37)
Siripongsatian (48)	12	12.5	0.5	6.5	6.5	0.46 (0.17 – 0.75)	31	31.5	0.5	11.5	20.5	0.62 (0.45 – 0.80)
Lan (41)	15	15.5	0.5	12.5	3.5	0.19 (−0.03 – 0.41)	16	16.5	0.5	13.5	3.5	0.18 (−0.03 – 0.38)
Shi (47)	2	2.5	0.5				3	3.5	0.5			
Zhang (51)	3	3.5	0.5				5	5.5	0.5			
**Overall**						**0.27 (0.11 – 0.43)**						**0.28 (0.08 – 0.48)**

**Pancreatic cancer**	**Patient-based analysis**						**Lesion-based analysis**					
	**Number of patients**	**FAPI PET**		**[18F]FDG PET**		**Risk Difference (95% CI)**	**Number of lesions**	**FAPI PET**		**[18F]FDG PET**		**Risk Difference (95% CI)**
**Author**		**positive event**	**negative event**	**positive event**	**negative event**			**positive event**	**negative event**	**positive event**	**negative event**	
Çermik (26)	2	2.5	0.5	2.5	0.5	0.00 (−0.60 – 0.60)						
Chen (27)							4	3	1	3	1	0.00 (−0.60 – 0.60)
Lan (40)							7	5	2	5	2	0.00 (−0.47 – 0.47)
Pang (44)	7	7.5	0.5	6.5	1.5	0.12 (−0.19 – 0.44)	7	7.5	0.5	6.5	1.5	0.12 (−0.19 – 0.44)
Pang (32)	26	26.5	0.5	19.5	7.5	0.26 (0.08 – 0.44)	26	26.5	0.5	19.5	7.5	0.26 (0.08 – 0.44)
Zhang (52)	30	30.5	0.5	30.5	0.5	0.00 (−0.06 – 0.26)	30	30.5	0.5	30.5	0.5	0.00 (−0.06 – 0.06)
**Overall**						**0.10 (−0.06 – 0.26)**						**0.09 (−0.06 – 0.24)**

Abbreviations: FAPI: fibroblast activation protein inhibitor, PET: positron emission tomography, [18F]FDG: 2-deoxy-2-[18F]fluoro-D-glucose, CI: confidence interval

Supplemental Table S7. Complete data of positive detection rate analysis (risk difference) between radiolabelled FAPI and [^{18}F]FDG PET imaging for metastases.

**Liver metastases of gastrointestinal origin**	**Patient-based analysis**						**Lesion-based analysis**					
	**Number of patients**	**FAPI PET**		**[^{18}F]FDG PET**		**Risk Difference (95% CI)**	**Number of lesions**	**FAPI PET**		**[^{18}F]FDG PET**		**Risk Difference (95% CI)**
**Author**		**positive event**	**negative event**	**positive event**	**negative event**			**positive event**	**negative event**	**positive event**	**negative event**	
Zhang (52)	5	5.5	0.5	5.5	0.5	0.00 (−0.31 – 0.31)	256	256.5	0.5	181.5	75.5	0.29 (0.24 – 0.35)
Li (42)	10	9	1	8	2	0.10 (−0.21 – 0.41)	25	24	1	21	4	0.12 (−0.04 – 0.28)
Lin (43)	9	9.5	0.5	9.5	0.5	0.00 (−0.19 – 0.19)	30	30.5	0.5	16.5	14.5	0.45 (0.27 – 0.63)
Gündoğan (38)	5	5.5	0.5	5.5	0.5	0.00 (−0.31 – 0.31)	188	188.5	0.5	148.5	40.5	0.21 (0.15 – 0.27)
Lan (41)							65	65.5	0.5	60.5	5.5	0.08 (0.01 – 0.15)
Elboga (37)							37	37.5	0.5	28.5	9.5	0.24 (0.09 – 0.38)
Şahin (45)							98	94	4	78	20	0.16 (0.07 – 0.25)
Wu (50)							48	42	6	16	32	0.54 (0.38 – 0.70)
Koerber (34)							14	14.5	0.5			
**Overall**						**0.02 (−0.11 – 0.15)**						**0.25 (0.15 – 0.36)**

**Lymph node metastases of primary HPB tumours**	**Patient-based analysis**						**Lesion-based analysis**					
	**Number of patients**	**FAPI PET**		**[^{18}F]FDG PET**		**Risk Difference (95% CI)**	**Number of lesions**	**FAPI PET**		**[^{18}F]FDG PET**		**Risk Difference (95% CI)**
**Author**		**positive event**	**negative event**	**positive event**	**negative event**			**positive event**	**negative event**	**positive event**	**negative event**	
Guo (29)							32	32.5	0.5	21.5	11.5	0.33 (0.17 – 0.50)
Siripongsatian (48)							47	47.5	0.5	34.5	13.5	0.27 (0.14 – 0.40)
Pang (32)							22	18	4	13	9	0.23 (−0.03 – 0.49)
Zhang (52)							43	43.5	0.5	30.5	13.5	0.30 (0.16 – 0.44)
Lan (40)							40	40.5	0.5	35.5	5.5	0.12 (0.01 – 0.23)
**Overall**												**0.24 (0.15 – 0.33)**

**Extra-hepatic distant metastases of primary HPB tumours**	**Patient-based analysis**						**Lesion-based analysis**					
	**Number of patients**	**FAPI PET**		**[18F]FDG PET**		**Risk Difference (95% CI)**	**Number of lesions**	**FAPI PET**		**[18F]FDG PET**		**Risk Difference (95% CI)**
**Author**		**positive event**	**negative event**	**positive event**	**negative event**			**positive event**	**negative event**	**positive event**	**negative event**	
Guo (29)							64	64.5	0.5	55.5	9.5	0.14 (0.05 – 0.23)
Siripongsatian (48)							27	27.5	0.5	24.5	3.5	0.11 (−0.02 – 0.24)
Pang (32)							141	129	12	62	79	0.48 (0.38 – 0.57)
Lan (40)							99	99.5	0.5	78.5	21.5	0.21 (0.13 – 0.29)
**Overall**												**0.23 (0.07 – 0.40)**

Abbreviations: FAPI: fibroblast activation protein inhibitor, PET: positron emission tomography, [18F]FDG: 2-deoxy-2-[18F]fluoro-D-glucose, CI: confidence interval

Supplemental Table S8. The pooled analysis of the positive detection rate of radiolabelled FAPI and [^{18}F]FDG PET imaging of primary HPB tumours and metastases.

Patient-based analysis		HCC	BTC	PC	Liver metastases *	LN metastases	Extra-hepatic distant metastases
**FAPI PET**	**Detection rate (95% CI)**	0.94 (0.87 − 0.97)	0.92 (0.79 − 0.97)	0.96 (0.85 − 0.99)	0.93 (0.83 − 0.98)		
**[^{18}F]FDG PET**	**Detection rate (95% CI)**	0.61 (0.48 − 0.72)	0.66 (0.47 − 0.80)	0.85 (0.62 − 0.95)	0.78 (0.64 − 0.88)		
**Lesion-based analysis**							
**FAPI PET**	**Detection rate (95% CI)**	0.97 (0.88 − 1.00)	0.93 (0.84 − 0.97)	0.91 (0.71 − 0.98)	0.98 (0.94 − 0.99)	0.97 (0.87 – 0.99)	0.98 (0.89 – 1.00)
**[^{18}F]FDG PET**	**Detection rate (95% CI)**	0.58 (0.48 − 0.67)	0.64 (0.43 − 0.81)	0.78 (0.64 − 0.87)	0.74 (0.59 − 0.84)	0.71 (0.62 – 0.79)	0.76 (0.53 – 0.90)

Abbreviations FAPI: fibroblast activation protein inhibitor, PET: positron emission tomography, [^{18}F]FDG: 2-deoxy-2-[^{18}F]fluoro-D-glucose, CI: confidence interval, HCC: hepatocellular carcinoma, BTC: biliary tract cancer, PC: pancreatic cancer, LN: lymph node, *: liver metastases of gastrointestinal origin

Supplemental Table S9. Complete data of the maximum standardized uptake value (SUVmax) of radiolabelled FAPI and [^{18}F]FDG PET imaging.

Hepatocellular carcinoma

	**FAPI PET**			**[^{18}F]FDG PET**		
**Author**	**N**	**Mean SUVmax**	**SD**	**N**	**Mean SUVmax**	**SD**
Çermik (26)	1	13.2	0	1	9.3	0
Guo (29)	15	12.16	4.71	11	5.66	2.37
Shi (46)	16	8.47	4.06	16	4.86	3.58
Wang (49)	30	6.96	5.01	20	5.89	3.38
Siripongsatian (48)	14	11.17	11.46	6	10.71	18.94
Zheng (33)	4	2.49	0.74			
Shi (47)	15	7.78	3.84			
Zhang (51)	33	8.5	5.5			

Biliary tract cancer

	**FAPI PET**			**[^{18}F]FDG PET**		
**Author**	**N**	**Mean SUVmax**	**SD**	**N**	**Mean SUVmax**	**SD**
Chen (36)	1	10.29	0	1	1.78	0
Lan (40)	3	16.07	2.72	3	9.73	6.97
Hirmas (30)	8	13.2	NR	8	7.5	NR
Guo (29)	7	16.14	5.45	4	5.58	4.11
Shi (46)	4	14.14	2.2	4	9.19	3.6
Siripongsatian (48)	31	18.45	19.41	11	10.5	16.84
Lan (41)	16	10.3	5.5	13	6.9	4.2
Zheng (33)	3	12.66	12.57			
Dendl (28)	4	14.7	NR			
Shi (47)	3	13.55	2.34			
Zhang (51)	5	7	6.6			

Pancreatic cancer

Author	FAPI PET			[18F]FDG PET		
	N	Mean SUVmax	SD	N	Mean SUVmax	SD
Çermik (26)	2	28.15	14	2	13.05	3.65
Chen (27)	4	20.6	10.23	4	4.74	1.69
Chen (36)	1	24.76	0	1	2.7	0
Lan (40)	7	14.49	9.17	7	3.75	2.11
Pang (44)	7	14.85	4.8	6	6.03	1.99
Hirmas (30)	56	13.2	NR	56	6.1	NR
Pang (32)	26	22.33	5.88	19	5.7	2.33
Zhang (52)	30	12.58	4.44	30	8.78	3.8
Zheng (33)	6	11.41	5.05			
Röhrich (35)	19	13.37	5.45			

Liver metastases of gastrointestinal origin

Author	FAPI PET			[18F]FDG PET		
	N	Mean SUVmax	SD	N	Mean SUVmax	SD
Pang (32)	74	7.93	1.9	15	4.1	1.09
Zhang (52)	104	4.16	1.89	104	7.63	2.15
Elboga (37)	37	18	12.94	28	6.05	3.48
Pang (31)	53	12.23	4.75	31	6.94	3.18
Li (42)	24	10.88	4.16	21	9.39	4.5
Kömek (39)	45	6.15	4.01	45	9.66	5.48
Lin (43)	30	5.43	2.57	16	5.4	2.09
Gündoğan (38)	5	6.6	2.7	5	8.1	2.7
Shi (47)	10	7.67	4.56			
Koerber (34)	14	9.54	3.74			

Lymph node metastases originating from primary HPB tumours

Author	FAPI PET			[18F]FDG PET		
	N	Mean SUVmax	SD	N	Mean SUVmax	SD
Guo (29)	32	11.89	6.68	21	4.58	2.9
Siripongsatian (48)	47	9.28	4.83	34	9.68	7.55
Pang (32)	45	9.63	3.52	23	3.3	1.5
Zhang (52)	29	8.65	5.01	29	7.7	3.76
Lan (41)	8	9.5	4.4	8	8.2	5.7
Röhrich (35)	6	14.13	8.5			

Extra-hepatic distant metastases originating from primary HPB tumours

Author	FAPI PET			[18F]FDG PET		
	N	Mean SUVmax	SD	N	Mean SUVmax	SD
Guo (29)	64	8.92	5.72	55	3.92	3.04
Siripongsatian (48)	27	13.05	10.6	24	5.28	3.77
Pang (32)	165	13.18	6.35	55	4.35	1.8
Lan (41)	12	10	4	12	7.3	5.7
Röhrich (35)	16	7.34	2.48			

Abbreviations: FAPI: fibroblast activation protein inhibitor, PET: positron emission tomography, [^{18}F]FDG: 2-deoxy-2-[^{18}F]fluoro-D-glucose, SUVmax: maximum standardized uptake value, SD: standard deviation, NR: not reported

Supplemental Table S10. Complete data of the target-to-background ratio (TBR) of radiolabelled FAPI and [^{18}F]FDG PET imaging.

Hepatocellular carcinoma

	**FAPI PET**			**[^{18}F]FDG PET**		
**Author**	**N**	**Mean TBR**	**SD**	**N**	**Mean TBR**	**SD**
Guo (29)	15	5.37	2.71	11	1.87	1.02
Shi (46)	16	7.13	5.52	16	2.39	2.21
Wang (49)	30	11.9	8.35	20	3.14	1.59
Siripongsatian (48)	14	7.82	9.48	6	3.39	5.44
Shi (47)	15	9.78	6.97			
Zhang (51)	33	5.5	3.7			

Biliary tract cancer

	**FAPI PET**			**[^{18}F]FDG PET**		
**Author**	**N**	**Mean TBR**	**SD**	**N**	**Mean TBR**	**SD**
Guo (29)	7	6.67	3.1	4	2.07	1.68
Shi (46)	4	26.46	4.94	4	4.42	19.4
Siripongsatian (48)	31	19.95	24.55	11	3.4	5.73
Shi (47)	3	26.92	4.68			
Zhang (51)	5	6.1	8.3			

Liver metastases of gastrointestinal origin

	**FAPI PET**			**[^{18}F]FDG PET**		
**Author**	**N**	**Mean TBR**	**SD**	**N**	**Mean TBR**	**SD**
Li (42)	24	7.24	2.83	21	2.92	0.93
Wu (50)	42	4.08	2.45	16	2.15	1.39
Kömek (39)	45	4.17	3.41	45	6.31	4.09
Lin (43)	30	4.15	1.47	16	2.28	1.16
Gündoğan (38)	5	4.8	3	5	3.2	1.2
Koerber (34)	14	11.36	NR			
Shi (47)	10	17.87	10.61			

Abbreviations: FAPI: fibroblast activation protein inhibitor, PET: positron emission tomography, [^{18}F]FDG: 2-deoxy-2-[^{18}F]fluoro-D-glucose, TBR: target-to-background ratio, SD: standard deviation, NR: not reported
